# Mesenchymal Stem/Stromal Cells in Stromal Evolution and Cancer Progression

**DOI:** 10.1155/2016/4824573

**Published:** 2015-12-21

**Authors:** Francesca Cammarota, Mikko O. Laukkanen

**Affiliations:** IRCCS SDN, Via Emanuele Gianturco 113, 80431 Naples, Italy

## Abstract

The study of cancer biology has mainly focused on malignant epithelial cancer cells, although tumors also contain a stromal compartment, which is composed of stem cells, tumor-associated fibroblasts (TAFs), endothelial cells, immune cells, adipocytes, cytokines, and various types of macromolecules comprising the extracellular matrix (ECM). The tumor stroma develops gradually in response to the needs of epithelial cancer cells during malignant progression initiating from increased local vascular permeability and ending to remodeling of desmoplastic loosely vascularized stromal ECM. The constant bidirectional interaction of epithelial cancer cells with the surrounding microenvironment allows damaged stromal cell usage as a source of nutrients for cancer cells, maintains the stroma renewal thus resembling a wound that does not heal, and affects the characteristics of tumor mesenchymal stem/stromal cells (MSCs). Although MSCs have been shown to coordinate tumor cell growth, dormancy, migration, invasion, metastasis, and drug resistance, recently they have been successfully used in treatment of hematopoietic malignancies to enhance the effect of total body irradiation-hematopoietic stem cell transplantation therapy. Hence, targeting the stromal elements in combination with conventional chemotherapeutics and usage of MSCs to attenuate graft-versus-host disease may offer new strategies to overcome cancer treatment failure and relapse of the disease.

## 1. Introduction

Tumors are organ-like structures [1] composed of numerous cell types whose interactions are required to drive and promote their growth and metastasis [2, 3]. Carcinogenic cells recruit nontumorigenic cells both locally from the neighboring tissues as well as from the circulation to construct the tumor microenvironment, which through reciprocal cancer-stroma interactions coevolves to promote cancer progression through paracrine signaling and physical interactions [4–8]. The tumor microenvironment contains cancer-associated fibroblasts (CAFs) [2], endothelial cells [9, 10], immune cells [11, 12], adipocytes [13], cancer stem cells (CSC) that differentiate into metastatic epithelial cells [14, 15], mesenchymal stem/stromal cells (MSCs) that can differentiate into fibroblasts and other types of cells representing mesenchymal lineages [16], and various types of extracellular matrix (ECM) proteins [3] needed for reciprocal messaging and the stimulation of tumor growth. The stroma, especially MSCs and stromal cells originating from MSCs, has recently been recognized as a player in carcinogenesis, affecting tumor growth, development, and progression beginning at the early steps of tumorigenesis [4] and influencing the construction of the microenvironment, epithelial mesenchymal transition, and metastasis, that is, functions that are essential for tumor maintenance and metastasis to other tissues [17–21].

## 2. Evolution of the Tumor Stroma

Simultaneous with the changes causing the immortalization of epithelial cells, there is a gradual evolution of the tumor microenvironment that includes (i) increased local vascular permeability; (ii) the extravasation of plasma and macromolecules, such as fibrinogen and plasminogen; (iii) the activation of coagulation mechanisms in the developing tumor microenvironment; (iv) the formation of fibrin gel deposits; (v) the formation of a provisional stroma comprising cancer cells, fibroblasts, and immune cells; (vi) the initiation of angiogenesis in the provisional stroma; (vii) the degradation and replacement of the provisional stroma fibrin with highly vascularized granulation connective tissue; (viii) the transformation of the stroma to desmoplastic, loosely vascularized, and dense connective tissue; and (ix) the remodeling of the stromal ECM, inducing local cancer cell migration and metastasis [22–26].

### 2.1. Increased Vascular Permeability

MSCs may contribute to increased vascular permeability alone or by attracting mast cells that are able to both initiate and sustain cellular trafficking. Increased vascular endothelial growth factor-A (VEGF-A) production is one of the main drivers of vascular hyperpermeability [27, 28]. VEGF-A binding to VEGF receptor 2 (VEGFR2) induces a conformational change and subsequent dimerization of the receptor, leading to autophosphorylation and initiation of downstream signal transduction [29]. The activated signal transduction leads to increased vascular permeability through two alternative mechanisms: by the synthesis of transcellular endothelial pores or by the transient opening of paracellular endothelial junctions. The action of VEGF depends on reactive oxygen and nitrogen species (ROS, RNS), the activation of the SRC family of protooncogenes, and their contact with adherens junction VE-cadherin proteins [28, 30]. According to recent reports, mast cells contribute to vascular permeability by secreting histamine, serotonin, and platelet-activating factor that activate TR3/Nur77 orphan nuclear transcription factor signaling. TR3/Nur77 increases vascular permeability by suppressing the expression of endothelial cell adherent junction-associated proteins (VE-cadherin, *β*-catenin, *γ*-catenin, and p120) and tight junction protein CLAUDIN 5 that maintains vascular homeostasis [31, 32]. Alternatively, mast cell secreted histamine induces vascular permeability by nitric oxide- (NO-) dependent vascular dilation and PKC/ROCK/NO-dependent endothelial barrier disruption or by binding to H1 G-protein coupled receptor that activates endothelial cell calcium influx enhancing vascular permeability related signal transduction [33, 34].

### 2.2. Development of the Fibrin Matrix-Derived Provisional Stroma into Mature Stroma

The extravasation of plasma components, such as fibrinogen and clotting proteins (prothrombin and factors V, VII, X, and XIII), through the endothelial cell layer is one of the earliest changes in the precancerous lesion environment and initiates a tissue response similar to wound healing [22, 24, 27, 34, 36–40]. The synthesis of fibrin from fibrinogen by the action of thrombin demarcates the first milestone in tumor stroma development [22, 27, 36, 39, 41, 42]. Thrombin cleaves fibrinogen into soluble fibrin monomers and activates the clotting factor XIII to factor XIIIa, which then converts the soluble fibrin monomers into insoluble fibrin polymers to form a cross-linked fibrin matrix [43]. The formation of the fibrin matrix dramatically changes the tissue composition by creating a gel that absorbs and arrests plasma solutes, resulting in tissue edema. Therefore, the fibrin gel forms an initial “provisional” stroma where the parenchymal tumor cells, mesenchymal stromal cells, and hematopoietic inflammatory cells can migrate to comprise the final tumor microenvironment [44]. Fibrin gel itself enhances angiogenesis, another early tumor stroma phenomenon, by protecting angiogenic growth factors from degradation, by inducing the production of proangiogenic molecules, and by directly creating angiogenic factors, such as fragment E [45]. The next phase in the development of the tumor microenvironment and the formation of the mature tumor stroma is a result of the coordinated actions of infiltrated macrophages and fibroblast-derived proteases that degrade the “provisional” stroma fibrin, replacing it with loose connective tissue [22, 39]. The loose connective tissue, which resembles the granulation tissue in healing wounds, stimulates the growth of fibroblasts and new blood vessels.

### 2.3. Development of Stromal Desmoplasia

One of the histological cornerstones of cancer development is the formation of a dense fibrotic stromal matrix comprising ECM and activated fibroblasts (myofibroblasts). In this last phase of stromal development, the granulation tissue transforms into desmoplastic dense connective tissue characterized by poor vascularization. The activation of the stroma, desmoplasia, can be interpreted as an attempt by the tumor tissue to heal the injury produced by the infiltrative and destructive growth of cancer cells, indicating the invasive and malignant characteristics of the tumor. However, it has been suggested that the increased collagen synthesis in desmoplasia, together with myofibroblast-induced tissue retraction, may paradoxically constitute a protective mechanism with invasive characteristics [10]. Mechanistically, the desmoplastic response is a poorly understood process associated with invasive tumors, such as diffuse infiltrative pancreatic and gastric carcinomas and infiltrating ductal (scrirrhous) carcinomas of the breast, and involves the excessive production of types I and III collagens and elastin [10]. In scrirrhous carcinomas of the breast, myofibroblasts, together with fibrin and collagen III, are mostly present in the immature mesenchymal stroma at the tumor periphery, while collagen-I is expressed in the mature sclerotic connective tissues of the tumor center [11].

In several types of tumors, such as thyroid cancer, the desmoplastic stromal reaction is a relatively common phenomenon, being present in up to 80% of medullary thyroid cancers and correlating with lymph node metastasis [46]. Indeed, the activated stroma has been considered a marker of invasion and metastatic cancer development. In thyroid cancers, desmoplasia induces increased production of collagen by stromal fibroblasts more prominently in anaplastic thyroid and medullary thyroid cancer than in papillary thyroid cancer, directly correlating with increased aggressiveness and lymph node metastasis [46]. It is therefore used as an intraoperative prognostic marker [47, 48]. The desmoplastic stroma in thyroid cancer contains activated fibroblasts (cancer-associated fibroblasts (CAFs) or myofibroblasts) that, together with the other stromal components, initiate the remodeling of the extracellular matrix [49]. ROS have been shown to promote the activation of these fibroblasts, which then increase tumor cell proliferation, tumor-associated inflammation, and angiogenesis by expressing invasion-associated factors and enzymes, such as fibroblast activation protein *α* (FAP*α*) and matrix metalloproteases (MMP), which are able to degrade and remodel the ECM [50–52].

### 2.4. Remodeling of the Stromal ECM Facilitates the Migration of Cancer Cells

The remodeling of the ECM induces the migration, invasion, and metastasis of cells by stimulating epithelial cell transformation and local migration by altering the cross-linked stromal structure. It therefore represents a critical element of cancer progression. Proteolytic degradation of the ECM affects the integrin-mediated anchorage of the cells, focal adhesions at cell membranes, cellular cytoskeletal organization, and the signal transduction regulating these structures. One of the main nonreceptor tyrosine kinases affected during this process is focal adhesion kinase (FAK), which directly signals through the SRC oncogene family kinases, linking integrin signaling to RAS-BRAF-MEK-ERK mitogen pathway signaling, thus inducing the malignant progression of tumor cells [53, 54].

## 3. MSCs and CAFs in the Tumor Stroma

In tumors, the MSCs either differentiate or maintain their primitive phenotype, thus participating in the construction of the stroma and supporting cancer cell growth through their secretion of cytokines and growth factors [55–58]. The source of tumor MSCs has not been completely clarified, although they have been isolated from most tissues [59], suggesting that they have a local origin. Another potential source is the circulating bone marrow-derived MSCs that extravasate from the circulation and then home and engraft onto growing tumors [60–62]. Evidence of the tumor tropism of MSCs has been obtained from* in vivo* studies, in which transplanted MSCs were demonstrated to migrate to tumors [60]. Interestingly, the migration of MSCs to tumors seems to be increased by cell damage, which typically characterizes the clinical treatments used for cancer, such as radiation therapy, possibly due to the increased cytokine and chemokine secretion from the injured tissues [61]. Although the detailed mechanism(s) underlying the migration and homing of MSCs to tumors are not well documented, MSCs have a similar homing mechanism to inflammatory cells, hematopoietic stem cells, and cancer cells in that they utilize the same adhesion molecules and cytokines/chemokines, most notably the CXCR4-CXCL12 receptor-ligand binding system [63, 64].

Cancer-associated fibroblasts (CAFs) are derived from both mesenchymal stem/stromal cells and local fibroblasts [60, 65], thus supporting observations suggesting the presence of primitive undifferentiated MSCs even in advanced cancers. MSCs and CAFs are both heterogeneous populations that may have different phenotypic characteristics even within the same type of cancer [66, 67]. CAFs are a rich source of growth promoting molecules (e.g., HGF, LOXL2, and TENASCIN-C) and proangiogenic factors (e.g., VEGF), hence playing an important role in cancer progression and metastasis [65] by stimulating epithelial mesenchymal transition (e.g., by TWIST1 and SNAIL production), by causing epigenetic changes, and by altering three-dimensional structure of ECM (e.g., by MMP and plasminogen activator protein production) [67, 68]. CAFs are known to support survival and proliferation of cancer cells in metastasis in a similar mechanism as in primary tumor. An interesting characteristic of CAFs is their ability to migrate together with epithelial cancer cells, thus suggesting a role in the intravasation and extravasation of epithelial cells in metastasis process by promoting cancer cell transmigration through endothelial cell layers [69, 70], hence supporting the hypothesis that primary tumor may be able to facilitate metastasis by providing the microenvironment.

Previous papers have demonstrated that in certain cases CAFs, similarly with MSCs, maintain normal fibroblast tumor suppressive characteristics. Receptor-ligand ROBO1-SLIT2 cancer-stroma interaction has been shown to reduce tumor cell proliferation by reducing PI3K-*β*-catenin and SDF1-CXCR4 signal transduction and consequent cancer cell malignancy [71, 72]. Interestingly, aggression of cancer cells lacking ROBO1 receptor molecule was increased by CAFs expressing SLIT1 ligand [73], whereas RNAi SLIT1 increased hepatocyte growth factor-mediated cancer cell migration and invasion by upregulating CDC42 Rho GTPase activity [74], thus giving more insight into the inhibitory-stimulatory mechanism of CAFs that may depend on phenotypic differences of stromal cells and malignant epithelial cells. The study of Takahashi and coworkers corroborated selective tumor suppressor properties of CAFs. According to their observations, podoplanin positive CAFs predicted poor survival and outcome among lung adenocarcinoma and squamous cell carcinoma patients, whereas in small cell lung cancer podoplanin positivity suggested better prognosis [75]. While the characteristics of MSCs and CAFs are well documented in tumor support, more work is needed to study differentiation of MSCs to cancer-associated fibroblasts and tumor suppressor properties of these cell populations.

### 3.1. Tumor MSCs Differ from Normal MSCs

It is important to note that the MSCs and CAFs localized in the tumor stroma have a different phenotype compared to MSCs and fibroblasts isolated from normal tissues, which may be a result of the constant exposure of these cells to inflammatory and cancer cell-secreted cytokines inducing procancerous characteristics [76–78]. Although the tumor-associated MSCs share similar cell surface markers and functionality with normal tissue MSCs, the paracrine effect on cancer cell proliferation is different [58]. Whereas normal tissue MSCs have been suggested to reduce inflammatory and cancer cell proliferation [79, 80], tumor-associated MSCs increase their growth [53–55]. The phenotypic and functional modifications of MSCs are further supported by case reports demonstrating unusual papillary thyroid carcinoma- and lung carcinoma-associated intratumoral heterotrophic ossification [81, 82]. However, the heterotopic ossification caused by the differentiation of MSCs into osteoprogenitor cells and further differentiated cells [83] may also be the result of abnormal differentiation signaling originating from the surrounding tumor stroma.

### 3.2. MSCs Support Parenchymal Cells in the Tumor Microenvironment

Most transformed cell lines are not able to survive after transplantation and are therefore considered to be cells continuously growing without tumorigenic characteristics. Even among highly carcinogenic cell lines, only a small subset harboring stem cell-like characteristics are able to initiate tumor growth* in vivo* [84]. Due to their phenotype and functional properties, cells with clonal tumor-initiating capacity are called cancer stem cells (CSCs) or tumor-initiating cells (TICs). The CSCs/TICs reside in specific niches in the tumor microenvironment that maintain their plasticity, protect them from immune defense mechanisms, and modify their metastatic potential. MSCs have been shown to interact with CSCs/TICs, supporting parenchymal cell growth and causing increased resistance to therapy [35], cancer cell dormancy, and evasion from the immune system [85] either through paracrine secretion [35, 57] or gap junction contact [85]. Alternatively, MSCs can affect epithelial cancer cell function by direct contact, causing increased expression of microRNAs, such as mir199a and stem cell-associated factors, in the epithelial cells [86]. MSCs secrete various growth-supporting cytokines, growth factors, and microRNAs that in some cases are stored inside extracellular vesicular particles (exosomes) [87]. Exosomes are small (40–100 nm in diameter) membrane-bound organelles that function as part of an intercellular communication mechanism. During tumorigenesis, exosome-bound factors have been demonstrated to modify the phenotype of the epithelial cancer cells or tumor stromal cells to support the aggressive phenotype and tumor progression. The exosomes characteristically include various types of molecules, including matrix metalloproteases [88], platelet-derived growth factors [89], molecules that activate signal transduction [90], oncomiRs, bioactive lipids, and metabolites [91].

The gap junctions between stromal cells and parenchymal cells are an important gateway for microRNA transfer. Interestingly, gap junction-transferred* CXCL12-*targeting microRNAs* mir127*,* mir197*,* mir222*, and* mir223*, can reduce cancer cell proliferation and even induce dormancy that may last for decades, eventually leading to a relapse of the disease due to bone marrow metastasis [91]. A recent study demonstrated that there was gap junction-mediated intercellular communication between bone marrow MSCs and primitive Oct4-expressing breast cancer tumor-initiating cells [91], suggesting that there is a preference for gap junction-mediated connections between cells harboring stem cell characteristics, also suggesting that these cells show similarities to hematopoietic stem cell dormancy [92, 93].

**Figure 1:**
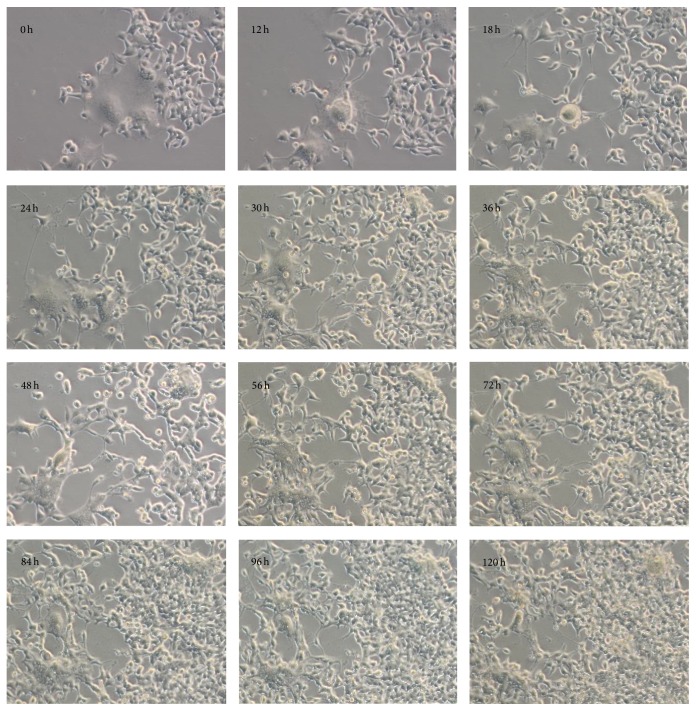
Time-lapse images of HEK 293T and bone marrow mesenchymal stem cell culture [35]. Apoptotic MSCs were surrounded and consumed by the HEK 293T cells during the 120-hour period.

**Figure 2:**
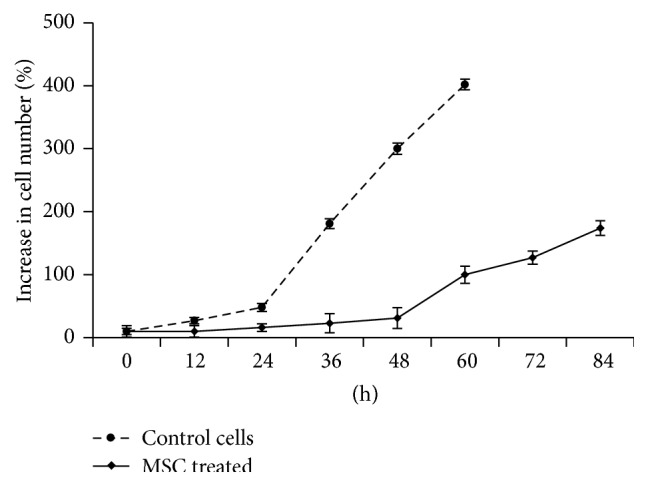
The results of cell count analyses of HEK 293T cells grown in the absence (control cells) or presence of apoptotic MSCs (MSC-treated) [35]. The analyses suggest that there was decreased growth of the MSC-treated cells during 0–48 hours of cytoplasmic material transfer from the MSCs to HEK 293T cells.

In addition to the gap junction structures, it has been suggested that breast cancer cells form hybrids with MSCs [94]. Recently, a number of publications have suggested that there is a direct connection between MSCs and cancer cells, resulting in increased epithelial cell growth and survival [55, 95–99]. The direct connection between cancer cells and stromal cells has been studied in a cancer cell-stromal cell coculture system that could itself promote cell-to-cell contacts. Characteristically, primary nontransformed cells avoided direct contact with other cells, limiting their growth and migration, a phenomenon known as contact inhibition [100]. We have studied the interaction of transformed cells and human bone marrow-derived mesenchymal stem cells and demonstrated that physical MSC-MSC or MSC-cancer cell interaction is mediated through temporal membrane protrusions, which direct the movement of the cells (Supplemental film 1 in Supplementary Material available online at http://dx.doi.org/10.1155/2016/4824573) [97]. Unlike primary cells, cancer cells have lost the contact inhibition phenotype [101] and therefore characteristically create network connections by reaching towards other cancer cells and nontransformed primary cells, such as MSCs (Supplemental film 2). The aggression of cancer cells in coculture can induce damage to the MSCs and activate apoptosis, with the consequent loss of MSCs locomotion [97]. The damaged stromal cells that are immobilized and unable to avoid contact are vulnerable to cancer cell aggression, resulting in material transfer from stromal cells to epithelial cancer cells (Figure 1) [97, 99]. Consequently, the tumor initiating and prometastasis effects of the transformed cells increase significantly, and this is linked to increased mitochondrial activity. Importantly, we demonstrated by serial dilution transplantation and a flow cytometric analysis of clusters of differentiation markers that the transformed cells did not show any stem cell characteristics, suggesting that increased metabolic activity mediates the aggressiveness of the epithelial cells. Interestingly, a cell-count analysis of the transformed cells suggested that there was only minor growth during cytoplasmic material transfer, whereas control cells grown in the absence of damaged MSCs showed continuous cell proliferation (Figure 2) [97]. Thus, these observations may suggest that cancer cells can remove injured and dying tumor cells, thereby temporally increasing their own growth characteristics. This hypothesis is corroborated by findings suggesting that there was increased mouse tumor cell growth in the presence of apoptotic cancer cells or fibroblasts, underlining the importance of cellular damage in direct cell-to-cell contact and the transfer of cellular macromolecules [55, 97, 102, 103].

In addition to the paracrine-mediated, gap junction-mediated, and direct contact-based support of tumorigenesis, stromal cells have been shown to affect the transcriptome of tumors, especially in patients with a relapse of the disease. The dataset analyses have demonstrated upregulation of the gene expression patterns associated with poor patient survival [19–21]. Analyses have shown that colon cancer patients had significantly higher expression levels of genes related to the risk of recurrence, such as* TGF-β*,* CALD1*,* POSTN*,* FAP*,* IGFBP7*, and* MPG*, in their stromal cells compared to their epithelial cancer cells [19, 20]. Notably, among all stromal cell populations, the CAFs showed the strongest expression level of the stem/serrated/mesenchymal transcription subtype of colorectal cancer [20]. Thus, the “stromal signatures” characteristic of different colorectal cancer subtypes may have clinical relevance and may even serve as a prognostic marker of the disease [20].

## 4. MSCs in Cancer and in Graft-versus-Host Disease Therapy

Although preclinical cancer therapy studies in MSC xenograft mouse models have given contradictory results, which may be due to model systems used in the experiments, timing of MSC transplantation, and protocol of propagating cells* ex vivo* [104], MSCs have maintained their therapeutic potential in cancer treatments. In ongoing phase I/II study (NCT02008539) autologous MSCs transduced with herpes simplex virus-thymidine kinase (*HSV-TK*) retrovirus are intravenously injected into patients with advanced gastrointestinal tumors followed by ganciclovir treatment to study safety and tolerability of the therapy [105]. Significantly more excitement has received the property of MSCs to enhance hematopoietic stem cell engraftment and to prevent graft-versus-host disease (GVHD). GVHD, the attack of transplanted immune cells against recipient tissues, is a frequent complication of autologous bone marrow transplantation in the treatment of hematologic malignancies. MSC transplantation has been demonstrated to be safe in a clinical pilot study in which twenty patients with hematologic malignancies received MSC transplantation together with allogeneic hematopoietic cell transfusion. The cotransplantation resulted in 10% one-year nonrelapse mortality, 80% overall survival, 60% progression-free survival, and 10% graft-derived death. Similar treatments without MSC cotransplantation have resulted in 37% one-year nonrelapse mortality, 44% overall survival, 38% progression-free survival, and 31% graft-derived death. In another clinical trial, the effect of MSC transfusion was studied in 75 patients with persistent acute GVHD that did not respond to immunosuppressive agents. The GVHD status before MSC treatments was improving in 2.7% of patients, was unchanged in 29.3% of patients, and was worsening or showing maximal GVHD level in 66.7% of patients. Serial MSC transfusions demonstrated an encouraging 61.3% overall response rate that was followed by 78.1% 100-day survival level, whereas patients who did not responded to MSC infusions had 100-day survival of only 31%. The* ex vivo* culture of MSCs before transfusion decreases the overall survival of the patients although the phase I/II clinical trials have demonstrated safety of the procedure [106–109]. Currently new clinical trials (EudraCT number 2006-004101-26, EudraCT number 2009-014980-38, NCT01763099, NCT01763086, and NCT01941394) are recruiting patients to further explore the ability of MSCs to prevent GVHD. Hence, based on the observed data and ongoing studies, MSCs transplantation is a promising tool to improve the efficiency of cancer treatments.

## 5. Tumor Stroma as a Drug Target and Mediator of Drug Resistance

Molecular-targeting drugs against activated oncogenes whose continues expression is essential for the survival of cancer cells, a phenomenon known as oncogene addiction, represent the latest development in cancer treatment. However, the efficacy of these therapies is reduced by the development of drug resistance. Cell autonomous drug resistance, also known as primary resistance, is caused by the constitutive activation (mutation) of signal transducers downstream of the targeted molecule or by the simultaneous activation of compensatory pathways. Secondary or acquired resistance is observed when neoplastic cells originally sensitive to molecular inhibitors lose their response to these drugs. This can occur by target reactivation or by compensatory bypass. The cell autonomous mechanism implies that there are secondary mutations in the oncogenic kinase that render it refractory to the treatment. In contrast, compensatory bypass involves the compensatory activation of alternative kinases, thereby reducing the biological effects of the drug [110].

Together with the cell autonomous mechanisms of resistance to molecular inhibitors, many studies have suggested that an important role is played by noncell autonomous signals, originated by the cellular components of the tumor microenvironment [110]. A striking feature of metastatic tumor cells is their ability to plastically adapt to diverse microenvironmental conditions and to overcome single-drug treatment. The mechanisms underlying this mode of drug resistance are largely elusive and have thus been the subject of intense preclinical investigation. Recent studies have highlighted a crucial role for the interaction between cancer cells and the tumor microenvironment, leading investigators to hypothesize a completely different and unconventional mechanism of resistance to molecular inhibitors [97, 99, 103]. Although these studies were conducted in coculture systems, they form a proof of principle, demonstrating a novel mechanism by which cancer cells can survive under extreme stress and even utilize the severe culture conditions to improve their growth characteristics.

Poorly vascularized, desmoplastic stroma supports tumorigenesis and simultaneously forms a barrier for chemotherapeutic drugs, making it as an attractive drug target. Combination treatments targeting both the stroma and cancer cells with cytotoxins have shown promising results in preclinical and clinical experiments. A complete depletion of stroma by IPI-926 and a sonic hedgehog inhibitor, together with gemcitabine administration, increased the drug delivery in a preclinical mouse model, thus underlining the importance of the stroma for tumor development and maintenance and further suggesting the efficacy of combination therapy that targets both cancer cells and tumor stromal components [111]. A clinical study with partial stromal depletion using CD40-activated macrophages as well as a preclinical work utilizing enzymatic depletion of ECM hyaluronan demonstrated improved patient survival and increased drug delivery into the tumor [112]. In addition to the destruction of the whole stroma, preclinical studies have shown reduced tumorigenesis after depletion of stroma-supporting myofibroblasts using antibodies targeted against fibroblast activation protein (FAP) [113]. Clinical studies have demonstrated safety of anti-FAP infusion into patients with colorectal carcinoma, metastatic colorectal cancer, and small cell lung cancer although no tumor response has been observed [114, 115]. Despite poor success in cancer therapy, a recent anti-FAP preclinical study of malignant pleural mesothelioma, an incurable disease resulting from exposure to asbestos, suggested lysis of FAP positive cells, inhibited tumor growth, and significantly prolonged the survival of the mice [116]. The data encouraged the research team from the University of Zurich Switzerland to open a new clinical phase I trial (NCT01722149) to study the effect of FAP-specific CD8 positive T cells in malignant pleural mesothelioma patients. In the trial the patients are infused with 1 × 10^6^ adoptively transferred FAP-specific retrovirally reprogrammed T cells directly in the pleural effusion to evaluate the safety and the efficacy of the immunotherapy. Besides antibodies FAP vaccination studies have resulted in some excitement in preclinical level demonstrating significantly reduced tumor growth and metastasis in B16/F10.9 melanoma, 4T1 breast cancer, and EL4 thymoma mouse models [117, 118]. Mechanistically the vaccination sensitizes fibroblasts to CD8 T-cell attack, which leads to decreased collagen production and the significantly increased uptake of chemotherapeutic drugs [119].

Stromal fibrosis can also be inhibited using antioxidants, such as high-dose vitamin E, which increase survival in rat models [120] or by targeting angiotensin II type 1 receptors and angiotensin-converting enzyme activity [121]. In an anaplastic thyroid cancer xenograft model, bevacizumab (Avastin) treatment reduced the macrophage infiltration and inflammatory cytokine expression. More importantly, bevacizumab treatment reduced vascular permeability, thus affecting the first step of tumor stroma development [122]. There have so far been no convincing clinical studies showing the efficacy of tumor stroma inhibition in the most aggressive forms of thyroid cancer. However, because there is no treatment for the anaplastic form of thyroid cancer, studies of combination therapies targeting both the cancer cells and stroma might offer clinically relevant protocols and treatment alternatives.

Collectively, the continuously developing tumor stroma that simultaneously contains different stromal developmental phases has the versatile ability to support epithelial cancer cell growth. Although the presence and function of mesenchymal stem cells in the tumor microenvironment is still incompletely understood, the current knowledge suggests a crucial role for MSCs in the construction of the microenvironment, nurturing CSC/TIC cells, and supporting differentiated epithelial cancer cells. Based on this knowledge, targeting the stromal components in combination with the cancer cells themselves may increase the efficacy of cancer therapy.

## Supplementary Material

Cell culture: Human bone marrow mesenchymal stromal cells (MSCs) and HEK 293T cells (ATCC, Manassas, VA, USA) were cultured in *α*-MEM 10% FBS *α*-MEM powder (Invitrogen cat#12000-014, Paisley, UK), defined “FBS” (HyClone cat#SH3007003M, Logan, UT, USA) supplemented with penicillin-streptomycin (Sigma, St Louis, MO, USA), glutamine (Invitrogen cat#25030024), and minimal essential amino acids, NEAA (Invitrogen cat#11140035). The *α*-MEM powder was dissolved to commercial cell culture level water (Sigma). Sodium carbonate (Sigma) (2.2g/l) was added before filtration. Condensed serum-free medium from confluent HEK 293T culture was added to the MSC. HEK 293T cells were used for co-culture because of their robust growth abilities and strong survival potential under severe cell stress. The treated MSCs (2x105 cells) were seeded with approximately 10-20 HEK 293T cells per 10-cm dish to allow the co-culture to continue for 4-6 passages. The dishes were passaged at 6-day intervals.
Growth curve analysis: For the growth curve analysis 10,000 cells were seeded on 6-well plates and counted in triplicate in 12-hour intervals.
Time-lapse imaging: Cells were grown in CO2-independent medium (Invitrogen) supplemented with 10% FBS, MEAA, penicillin streptomycin, and glutamine on prewarmed support for 7 days. Medium was refreshed on every 3 days. Cell growth and movements were monitored by time-lapse imaging (20x magnification) at 2-min intervals (Zeiss Axiovert 200M inverted microscope, Carl Zeiss, Oberkochen, Germany).

## Abbreviations

CAF: Cancer-associated fibroblast
CDC42: Cell division control protein 42 homolog
CSC: Cancer stem cell
CXCL12: Stromal derived factor 1 or SDF-1
CXCR4: C-X-C chemokine receptor type 4 or SDF-1 receptor
ECM: Extracellular matrix
FAK: Focal adhesion kinase
FAP: Fibroblast activation protein
GVHD: Graft-versus-host disease
HGF: Hepatocyte growth factor
HSV-TK: Herpes simplex virus-thymidine kinase
LOXL2: Lysyl oxidase-like 2
MMP: Matrix metalloproteinase
MSC: Mesenchymal stem cell
NO: Nitric oxide
PKC: Protein kinase C
ROBO1: Roundabout (axon guidance receptor)
ROCK: Rho-associated coiled-coil kinase
RNAi: RNA interference
ROS: Reactive oxygen species
RNS: Reactive nitrogen species
SLIT2: Slit homolog protein 2
SNAIL: Snail family zinc finger 1
TAF: Tumor-associated fibroblast
TIC: Tumor initiating cell
TWIST1: Twist family BHLH transcription factor I
VEGF: Vascular endothelial growth factor.
